# Coronary Arteries Lesions in Kawasaki Disease: Risk Factors in an Italian Cohort

**DOI:** 10.3390/biomedicines12092010

**Published:** 2024-09-03

**Authors:** Elisabetta Morana, Fiorentina Guida, Laura Andreozzi, Leonardo Frazzoni, Lucia Augusta Baselli, Francesca Lami, Elena Corinaldesi, Cristina Cicero, Lorenzo Mambelli, Barbara Bigucci, Andrea Taddio, Chiara Ghizzi, Michela Cappella, Paola Fernicola, Marcello Lanari, Rocco Maurizio Zagari, Marianna Fabi

**Affiliations:** 1 Specialty School of Paediatrics, Alma Mater Studiorum, University of Bologna, 40126 Bologna, Italy; elisabetta.morana@studio.unibo.it; 2 Department of Medical and Surgical Sciences, Alma Mater Studiorum, University of Bologna, 40126 Bologna, Italy; fiorentina.guida@unibo.it (F.G.); marcello.lanari@unibo.it (M.L.); 3 Pediatric Emergency Unit, IRCCS Azienda Ospedaliero-Universitaria di Bologna, 40138 Bologna, Italy; marianna.fabi@aosp.bo.it; 4 Gastroenterology and Digestive Endoscopy Unit, Forlì-Cesena Hospitals, AUSL Romagna, 47121 Forlì, Italy; 5 Pediatric Intermediate Care Unit, Fondazione IRCCS Ca’ Granda Ospedale Maggiore Policlinico, Via della Commenda 9, 20122 Milan, Italy; lucia.baselli@policlinico.mi.it; 6 Department of Medical and Surgical Sciences for Mothers, Children and Adults, University of Modena and Reggio Emilia, 41125 Modena, Italy; lami.francesca@aou.mo.it; 7 Pediatric Unit, Carpi Hospital, 41012 Carpi, Italy; e.corinaldesi@ausl.mo.it; 8 Department of Pediatrics, AUSL, Guglielmo da Saliceto Hospital, 29121 Piacenza, Italy; c.cicero@ausl.pc.it; 9 Department of Paediatrics, Santa Maria delle Croci Hospital, AUSL della Romagna, 48121 Ravenna, Italy; lorenzo.mambelli@auslromagna.it; 10 Pediatric Clinic, Rimini Hospital, AUSL Romagna, 47923 Rimini, Italy; barbara.bigucci@auslromagna.it; 11 Institute for Maternal and Child Health, IRCCS “Burlo Garofolo” and University of Trieste, 34137 Trieste, Italy; andrea.taddio@burlo.trieste.it; 12 Pediatric Unit, AUSL Bologna, Ospedale Maggiore, 40133 Bologna, Italy; c.ghizzi@ausl.bologna.it; 13 Pediatrics Unit, Santa Maria Nuova Hospital, Azienda Unità Sanitaria Locale (AUSL)-Scientific Institute for Research and Healthcare (IRCCS) of Reggio Emilia, 42123 Reggio Emilia, Italy; michela.cappella@ausl.re.it; 14 Pediatrics Unit, G.B. Morgagni-L. Pierantoni Hospital, Azienda Unità Sanitaria Locale (AUSL) Romagna, 47121 Forlì, Italy; paola.fernicola@auslromagna.it; 15 Gastro-Esophageal Organic Disease Unit, IRRCS Azienda Universitaria-Ospedaliera di Bologna, 40138 Bologna, Italy; roccomaurizio.zagari@unibo.it

**Keywords:** Kawasaki disease, coronary artery lesions, risk factors, Caucasian cohort, aneurysms, thoracic chest pain, IVIG, Italian children

## Abstract

*Background*: Kawasaki disease (KD) is a systemic vasculitis of medium arteries, particularly involving coronary arteries. Coronary artery lesions (CALs) is the most serious complication in the acute stage, potentially leading to ischemic cardiomyopathy, myocardial infarction and sudden death. Environmental factors and genetic background contribute to individual susceptibility to develop CALs. The aim of this study was to define the risk factors for CALs in an Italian cohort. *Methods*: Data of KD patients from 10 Italian sites were registered into a REDCap database where demographic and clinical data, laboratory findings and coronary status were recorded. KD was diagnosed according to AHA definition. We used multiple logistic regression analysis to identify independent risk factors for CALs. *Results*: A total of 517 patients were enrolled, mainly Caucasians (83.6%). Presentation was complete in 321 patients (62.8%) and IVIG responsiveness in 360 (70%). CALs developed in 136/517 (26.31%). Gender, age, ethnicity, clinical presentation, fever duration, non-coronary cardiac events, Hb, albumin and CRP were significantly different between patients with and without CALs, while seasonality was not. Male gender, age < 18 months, Asian ethnicity, incomplete presentation and fever > 10 days were independent risk factors for CALs. *Conclusions*: Age younger than 18 months, incomplete KD and longer fever duration are risk factors for CALs. Asian ethnicity also represents a risk factor in our Italian Cohort.

## 1. Introduction

Kawasaki disease (KD) is an acute, self-limiting vasculitis, affecting small- and medium-sized arteries, which occurs predominantly in infants and children under five years of age [1].

It is the leading cause of acquired heart disease in children due to its predilection for the coronary arteries, whose vascular structure can be damaged, potentially leading to the development of coronary artery lesions (CALs), such as dilation, aneurysms and stenosis. Since aneurysms and stenosis alter the laminar blood flow, they cause endothelial damage and a predisposition to thrombosis, and thus ischemic heart disease and myocardial infarction. In adult patients under forty years of age, coronary artery aneurysms from KD account for 5% of acute coronary syndromes [2,3,4] and it is estimated that, by 2030, 1 in 1600 adults will have a history of KD and about 10% of them will have coronary aneurysms needing a continuous cardiovascular follow-up [5].

Despite over fifty years after its first description, the etiology of KD remains still unclear, but it is believed to be multifactorial, with genetic background and environmental factors being crucial. The genetic predisposition is supported by the peculiar geographical distribution, which is higher in Asian compared to non-Asian children. Family linkage and genome-wide association studies identified genes or gene regions associated with a major incidence of KD [6,7], CAL development [8] and response to treatment [9,10].

The risk factors for CALs described so far are shared in different geographic areas: male gender, younger age, Asian ethnicity, incomplete KD, unresponsiveness to standard therapy or late treatment [10,11,12,13]. Presenting gastrointestinal features is frequent and is reported to be associated with coronary aneurysms in a mostly Caucasian cohort [10,14,15].

Although smaller CALs usually regress and large and giant ones may undergo pseudo-normalization of the caliber over time [2], the internal elastic lamina of tunica intima of these vessels is disrupted and suffers permanent damage. A process of myofibroblastic proliferation starts, altering the endothelium and the vascular structure, potentially leading to serious complications, such as thrombosis, stenosis or rupture.

Thus, it is crucial to define those children at higher risk for CALs from early stages of the disease in order to implement preemptive strategies and improve outcomes.

Our aim was to investigate the risk factors for CALs in an Italian cohort of children diagnosed with KD.

## 2. Materials and Methods

### 2.1. Patients Selection and Data Review

A multicenter retrospective and prospective study was conducted, including 11 Italian hospitals (IRCCS Azienda Ospedaliero-Universitaria di Bologna; Fondazione IRCCS Ca’ Granda Ospedale Maggiore Policlinico, Milan; Department of Medical and Surgical Sciences for Mothers, Children and Adults, Modena and Reggio Emilia; Pediatric Unit, Carpi Hospital; Department of Pediatrics, AUSL, Guglielmo da Saliceto Hospital, Piacenza; Department of Pediatrics, Santa Maria delle Croci Hospital, Ravenna; Pediatric Clinic, Rimini Hospital; Institute for Maternal and Child Health, IRCCS “Burlo Garofolo”, Trieste; Ospedale Maggiore, Bologna; Pediatrics Unit, IRCCS Santa Maria Nuova Hospital, Reggio Emilia; Pediatrics Unit, Forlì).

Data of KD patients diagnosed between January 2000 to June 2023 were recorded in a REDCap database. Eligible patients were identified and entered in the REDCap database by each site’s principal investigators.

Site participation was reviewed and approved by the institutional review board (or equivalent) at each enrolling site according to local regulations including requirements for patient consent and/or dissent.

Patient data include patient demographics; clinical data, such as presenting signs and symptoms; serial laboratory and imaging investigations; and cardiac (coronary and non-coronary) data, treatment and complications. Laboratory exams and echocardiographic findings of the acute, defined as 1st to 10th days after fever onset, and subacute stages, defined as 11th to 20th after fever onset, were recorded in the dataset.

Data were reviewed at the Data Coordinating Center (IRCSS Azienda Ospedaliero-Universitaria di Bologna, Italy) to ensure patient eligibility and data completeness and accuracy.

### 2.2. Clinical Definitions

KD was diagnosed according to American Heart Association (AHA) guideline criteria: prolonged fever (>5 days), cutaneous rash, mucosal changes in oral cavity, bilateral non-exudative conjunctivitis, edema and erythema of extremities and bilateral, non-suppurative cervical lymphadenopathy [1].

Complete KD was defined as the presence of at least 4 criteria in addition to fever, while incomplete KD was defined when the clinical criteria were less than 4.

Late treatment was defined as the administration of the standard therapy after the 10th day of fever. When standard treatment was not administered, the patient was classified as “not treated” [1].

### 2.3. IVIG Treatment Protocols; IVIG Resistance

Patients were treated with high-dose (2 g/kg) intravenous immunoglobulins (IVIG) within the 10th day of fever, as soon as the diagnosis was made.

IVIG responsiveness was defined as defervescence within 48 h after the end of IVIG infusion, while IVIG resistance (or non-responsiveness) was defined as recrudescent or persistent fever 48 h after the end of IVIG infusion. Non-responder patients were treated with second line therapy, including a second dose of IVIG and adjunctive therapies, such as intravenous or oral steroid, and/or biologics, such as anti-IL1 and anti-TNF-α mono-clonal antibody therapy [1,6].

### 2.4. Laboratory Analysis

Complete blood count (including absolute and relative white blood cells count), hemoglobin levels (Hb), platelet count, serum glucose, hepatic and kidney function markers, acute phase reactant such as C-reactive protein (CRP) and erythrocytes sedimentation rate (ESR), serum albumin and serum electrolyte levels were performed on each patient at admission to the Pediatrics Emergency Department on the acute phase.

### 2.5. Cardiac Involvement

Serial echocardiography was also used to define coronary artery lesions (CALs) and non-coronary cardiac involvement, such as left ventricular acute dysfunction, pericardial effusion, mitral and/or aortic regurgitation.

Coronary artery involvement was defined from site-reported serial standardized echocardiographic measurements of the internal luminal dimension of the left main coronary artery (LMCA), proximal left anterior descending (LAD), left circumflex branch (LCx) and the proximal right coronary artery (RCA), with additional measurements of any coronary artery aneurysm.

Coronary artery dimensions were normalized for age and body surface area as Z-scores using published regression equations derived from a normal population [16].

CA involvement was then classified by Z-score as no involvement (Z < 2), dilation (Z from 2 to <2.5), small aneurysm (Z from 2.5 to <5), medium aneurysm (Z from 5 to 10) and large aneurysm (Z > 10), as per 2017 AHA guidelines [16].

The coronary status of each patient was assessed periodically until the 20th day from diagnosis.

Left ventricular dysfunction was defined when left ventricular ejection fraction (LVEF) was inferior to 55%; in particular, it was classified as mild dysfunction when comprising between 41% and 55%, moderate when between 31% and 40% and severe when ≤30%. LVEF was considered normal when between 56% and 78% [17,18].

### 2.6. Abdominal Involvement

The timing of the abdominal ultrasound screening was not standardized, nor described by a protocol, but left to the clinician’s judgment based on the clinical symptoms and the laboratory findings. Pathological findings were considered, including gallbladder hydrops, pancreatitis, free pelvic fluid into the pouch of Douglas and segmental bowel-wall thickening.

### 2.7. Statistical Analysis

Results are presented as absolute frequency and percentage for categorical variables and mean with standard deviation (SD) or median with interquartile range (IQR) for normally or non-normally distributed continuous variables, respectively. A multivariable logistic regression analysis was performed in order to identify variables independently associated with CALs. We investigated additional cut-off for risk factors for CALs based on results published by Son M.B.F. [12], that is, CRP > 13 mg/dL, Hb < 10.3 g/dL and serum albumin < 3.2 g/dL, linked to the persistence of CALs after the acute phase of the disease [12]. Odds ratios (ORs) and 95% confidence intervals (95% CIs) were estimated. A *p* < 0.05 was considered statistically significant. Analyses were conducted using STATA software version 16 (Stata Corp, College Station, TX, USA).

## 3. Results

Five hundred seventeen patients (mean age at diagnosis 42.07 ± 37.45 months; 316/517, 61.1% males) were enrolled. A total of 452 (87.4%) patients were Caucasian, 26 (5.0%) Asian, 22 (4.3%) Afro-American, and 17 (3.3%) of mixed ethnicities.

A total of 327 (63.2%) patients showed complete clinical presentation, 364 patients (70.4%) were IVIG responders and 41 patients (7.9%) were late treated.

CALs developed in 135 patients (26.1%), mostly males (93/135, 68.8%) at a mean age 36.5 ± 33.94 months.

Among CALs, 67 (49.7%) were aneurysms and 68 (50.3%) were ectasia (Table 1).

Among patients with CALs, aneurysms were more frequent in Asian children (12/14, 85.7%) than in non-Asians (55/122, 45.55%), in patients younger than 6 months of age (15/17, 88.21%) than in those older than 6 months (52/117, 44.47%), in patients below 18 months of age (27/46, 57.41% of CALs vs. 40/84, 47.67% in patients older than 18 months), in incomplete KD (39/61, 60.95% of CALs vs. 28/70, 39.41% of CALs in complete KD), in IVIG non-responders (17/26, 60.7% of CALs vs. 44/85, 53.06% of IVIG responders) and during summer (13/24, 54.2% of CALs vs. 54/110, 49.51% in other seasons) (Table 2).

A total of 124 (23.9%) patients developed non-coronary cardiac involvement: 27 (21.7%) of them showed left ventricular dysfunction, 48 (38.7%) patients had valvular regurgitation and 73 (58.9%) patients had pericardial effusion.

The comparison of demographics, clinical data and echocardiographic findings recorded during the acute stage of KD between patients who developed CALs and those without coronary involvement is shown in Table 3.

CALs were significantly more likely to develop in younger males, particularly those younger than 18 months. In addition, a correlation was found between ethnicity, incomplete clinical presentation, longer duration of fever and non-coronary cardiac involvement and the development of CALs. Regarding ethnicity, post hoc analysis revealed a statistically significant association with children of Asian ethnicity (*p* = 0.014). Among non-coronary cardiac findings, valvular regurgitation at the first cardiac evaluation was significantly more frequent in patients with CALs.

Patients younger than 18 months were more likely to be males and IVIG non-responders (respectively, *p* = 0.04 and *p* = 0.02). One-hundred and twenty of them (84.5%) showed non-coronary cardiac involvement.

Boys are younger than girls when they develop CALs (34.4 vs. 45 months, *p* = 0.0186). At the same time, female sex appears to be a protective factor for CAL development at younger age (21% with CALs versus 38% of boys, OR 0.44, *p* = 0.04).

No correlation was found between any clinical diagnostic signs (conjunctival hyperemia, alteration of extremities, mucositis, skin rash, lymphadenopathy) and CALs.

Abdominal ultrasound was performed on 132/517 (25.5%) patients. It showed pathological findings, such as gallbladder hydrops, pancreatitis, free pelvic fluid into the pouch of Douglas or segmental bowel-wall thickening, in 43 (32.6%) patients, and 14 of them (32.6%) developed CALs. Among them, only the circumferential thickening of the terminal ileum walls was significantly associated with CALs (*p* < 0.001).

Laboratory findings of the acute phase are shown in Table 4.

Among laboratory values, CRP > 13 mg/dL, serum albumin < 3.2 g/dL, Hb < 10.3 g/dL were significantly associated with CALs (respectively, *p* = 0.023, *p* = 0.014, *p* = 0.014) in the univariate analysis.

To assess which factor had the most significant effect on coronary outcome, we conducted a multivariate analysis using CALs as the outcome variable, with the following results.

**Figure 1:**
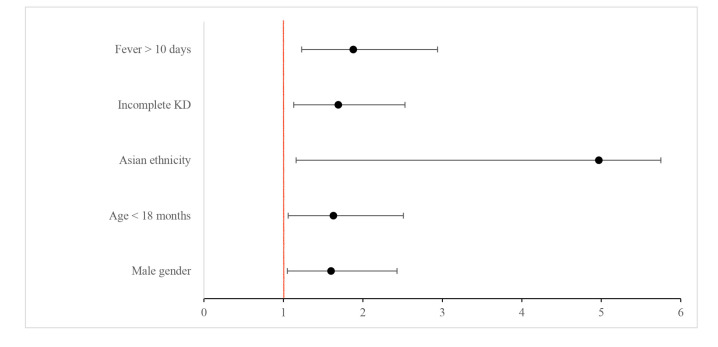
Independent risk factors for CALs. The red line is the line of no effect.

Among clinical variables, male gender, Asian ethnicity, incomplete KD, and fever duration >10 days were confirmed as independent risk factors for CAL development. Among laboratory variables, only serum albumin < 3.2 g/dL was significantly related to CALs (OR 1.77, CI 1.05–2.98), whereas Hb < 10.3 g/dL and CRP > 13 mg/dL were not (see Table 5 or Figure 1).

The other risk factors considered in the analysis, such as seasonality, age younger than 6 and 12 months and IVIG unresponsiveness, did not reach statistical significance in our cohort.

## 4. Discussion

In our Italian KD cohort, patients with CALs were more likely to be males, young, of Asian ethnicity, with incomplete presentation, longer fever duration and cardiac non-coronary ultrasound findings compared with those without coronary involvement. Among the blood test results, lower values of Hb and serum albumin and higher CRP values were more common in children with coronary involvement. In the multivariate analysis, male gender, age younger than 18 months, Asian ethnicity, incomplete clinical form and fever lasting more than 10 days were confirmed as independent risk factors for CALs.

These findings are in line with previous studies focusing on risk factors for coronary involvement in KD children.

Given that coronary artery involvement is the most serious complication of KD, heavily affecting the prognosis of these patients, the investigation of the risk factors for the development of CALs has captured the attention of clinicians and researchers since the discovery of KD. One of these pioneers was Asai in 1983, who tried to determine the indications for cardiac catheterization in these children, during a period in which echocardiography was not routinely used in the management of KD [19].

To date, the major risk factors associated with CALs have been reported to be as follows: male gender, Asian ethnicity, age younger than 12 months, incomplete clinical form, late treatment, longer fever duration, IVIG resistance, elevated CRP, anemia and hypoalbuminemia [1,13,20,21,22]. A recent systematic review and meta-analysis confirmed that male gender, IVIG resistance, late treatment and increased CRP levels are risk factors for CAL development in children with KD [23].

Firstly, our findings confirm that male gender is an independent risk factor for coronary lesions: Boys are more affected than their counterparts at any age, but they are globally younger than girls when they develop CALs. On the other hand, at younger age female sex appears to be a protective factor for CAL development, in line with previous studies [24].

Secondly, incomplete forms and longer duration of fever were risk factors for CALs in our cohort, as already shown in previous studies [1,14]. These two different risk factors could be two sides of the same coin. KD diagnosis is challenging for clinicians, especially in children with incomplete forms, who are at greater risk for late diagnosis and, therefore, longer duration of fever. In addition, these children may receive standard treatment later than those with complete forms, explaining the reason why late treatment is a well-known risk factor for CALs.

Not surprisingly, incomplete forms occur most commonly in younger children, who are at substantial risk of more severe KD with cardiac complications [1,11,12,13,25]. Notably, in our population, the age range at increased risk is slightly wider than previously documented [12,25]: CALs did not occur more frequently in children younger than 6 and 12 months, but in those younger than 18 months. Moreover, this age group is an independent risk factor. This can be partially explained by the peak incidence of KD diagnosis in Italy, namely in the second year of life [14]. Furthermore, a Japanese longitudinal national survey also showed that children with higher birth order are at risk for KD up to 18 months of age, probably due to greater exposure to pathogens from their older siblings and other childcare attendees [26].

Despite Asian patients accounting for only 5% of our cohort, they still proved themselves to be a high-risk population, in line with previous findings, supporting the role of genetic predisposition in causing more severe forms of disease. In our cohort, Asian ethnicity is not only the second most frequent one, but Asian children were more likely to develop CALs. In addition, among patients with CALs, aneurysms were more frequent in Asian than in non-Asian children. It is well known that Asian descendants have a higher incidence of KD than other race groups, but according to our findings, these children might also have a higher risk of severe coronary artery involvement, as already suggested by previous studies [27].

Our data show an incidence of coronary involvement during the acute stage similar to predominantly Caucasian cohorts. In a mixed Italian–French population, susceptibility to CALs is reported in 25% of patients [28] and, in a Spanish cohort, CALs developed in 23% of patients, in particular ectasia in 12%, while aneurysms in 9.6% of them, affecting up to 20% of children younger than 12 months [29]. Similarly, in a large cohort of Turkish children diagnosed with KD, CALs were found in 31.6% of patients CALs at the first echocardiographic assessment [30].

These percentages are higher compared to the last Japanese nationwide survey, showing 7.9% of Japanese KD children developing CALs. In addition, Japanese data documented coronary dilation in 5.6% of KD patients, aneurysms in 0.82%, giant coronary aneurysms in 0.13% and valvular lesions in 1.54% [31].

In contrast to these data, in our cohort, a high rate of coronary involvement was documented among Asian children. This discrepancy between our findings and Japanese data could be explained by several factors. Firstly, different rates of coronary involvement have been reported among various Asian populations, and our Asian subgroup included a wide range of origin countries (i.e., Japan, China, Korea, Vietnam) [32]. Secondly, the timing of treatment as the mean day of IVIG infusion could contribute to explain this discrepancy [20,33,34]. Although in our cohort only 8% of patients were late treated, Japanese studies [31] show a trend toward early and prompt treatment, with more than one-third of patients treated at day 5 from the onset of fever.

In addition, according to previous studies, non-coronary cardiac lesions, such as pericardial effusion, valve regurgitation and left ventricle dysfunction, are associated with CALs in both Caucasian and Asian children [35,36,37]. These associations were confirmed by our findings.

The occurrence of non-coronary cardiac events might be a sign of more intense systemic inflammation that results in an increased risk of CALs. According to this possible explanation, Printz et al. reported that left ventricular dysfunction and mitral regurgitation were associated with signs of inflammation in laboratory tests [36]. These findings are consistent with our results on laboratory tests, in which lower Hb and serum albumin values, higher CRP and the expression of more pronounced inflammation were significantly associated with CALs. Several studies reported these blood test abnormalities as risk factors for IVIG resistance or for coronary artery involvement [38,39,40].

Gastrointestinal symptoms are frequently presenting symptoms in KD [15] but their severity does not always require an abdominal ultrasound to be performed. In our cohort, we found that only the circumferential thickening of the terminal ileum walls was significantly associated with CALs. Segmental bowel thickening has already been reported as an additional diagnostic sign in KD [41]. Our findings suggest its potential role in predicting coronary involvement. The link between coronary artery and bowel walls could be an intense systemic vasculitis leading to multisystem signs, symptoms and complications.

Of note, IVIG unresponsiveness failed to reach statistical significance. Seasonality, potentially linked with environmental factors, was also not associated with coronary involvement.

Our findings lay the foundations to the development of a screening tool to identify children at higher risk of CALs at an earlier stage of KD.

An early identification of these children is essential, and it could be lifesaving. In fact, children with KD who develop CALs are at increased risk of cardiac events both during the acute phase and in later years, potentially leading to myocardial ischemia, myocardial infarction and sudden death [4,25,42,43,44].

Coronary involvement can vary from transient dilatations, in which it is not clear yet whether structural damage of the vessel occurs; to aneurysms, in which the destruction of the internal elastic lamina of tunica intima occurs [2,45,46]; and endothelial dysfunction [47,48,49,50,51] and blood hypercoagulability, potentially leading to stenosis, thrombosis and cardiac events. Increased megakaryocyte levels can also contribute to CAL development, affecting platelet function and, on the other side, together with monocytes, promoting cytokine production with the subsequent proliferation and migration of endothelial cells [51,52,53,54].

To date, several scoring systems have been developed to identify patients at higher risk of coronary involvement, but currently, a practical and effective screening tool in Caucasian population is not available or validated yet [10,12,55].

Machine learning algorithms seem to predict with high accuracy the occurrence of coronary lesions in KD [56,57,58,59], but these are not yet easily available for all medical institutions.

The prevention of CALs is a crucial point in the management of KD: identifying those patients at increased risk for severe disease means to have the possibility to personalize and intensify primary therapy in order to limit inflammation since earlier stages [60,61,62,63] and to apply a closer echocardiographic follow-up. In our population, Asian males younger than 18 months with incomplete presentation should be considered for adjunctive treatment and should be closely monitored especially when fever lasts more than 10 days.

Our study presents some strengths and limitations. KD is a disease characterized by a strong genetic predisposition and environmental influence, affecting both the incidence of the disease (that is 10 times lower in Western countries than Asian countries) and its clinical presentation, including coronary involvement [12,64]. Because of the low incidence of KD among Caucasian children, it is often difficult to conduct studies on KD with an adequately large sample. Our study is the first one investigating risk factors for coronary involvement in such a large cohort of Italian patients. According to the multicenter design, we were able to collect a considerable number of patients, considering the low incidence of KD in our country. This relatively large cohort can be considered a good representation of KD in the Italian population, reflecting local demographic and clinical characteristics, therapy and cardiac involvement. In addition, our results include variables that can be easily assessed with clinical examination and throughout medical history, leading to a prompt identification of patients at higher risk of CALs. On the other hand, the multicenter retrospective and prospective nature of this study limits the data homogeneity and completeness among participating centers, especially the laboratory and ultrasound findings. This discrepancy was the primary limitation for the use of these data in the multivariate model.

## 5. Conclusions

Our results show that high risk patients for CALs in Italian children with KD are boys, Asians, patients with incomplete presentation and long-lasting fever and patients up to 18 months of age. This age limit is slightly higher compared to those previously published.

## Figures and Tables

**Table 1:** Description of CALs among the cohort.

Variable	CALs (Tot = 135)
Ectasia, n (%)	68 (50.3%)
Aneurysms, n (%)	67 (49.7%)
Acute phase, n (%)	50 (74.6%)
Small aneurysms, n (%)	40 (80%)
Medium aneurysms, n (%)	9 (18%)
Giant aneurysms, n (%)	1 (2%)
Subacute phase, n (%)	17 (25.4%)
Small aneurysms, n (%)	3 (17.6%)
Medium aneurysms, n (%)	10 (58.8%)
Giant aneurysms, n (%)	4 (23.5%)

Legend: CALs stands for coronary artery lesions.

**Table 2:** The proportion of aneurysms among patients with CALs.

Variable	Aneurysms, n (%)
Asian children, n (%)	12 (85.7%)
Non-asian children, n (%)	55 (45.5%)
Age at onset < 6 months, n (%)	15 (88.2%)
Age at onset > 6 months, n (%)	52 (44.4%)
Age at onset < 18 months, n (%)	27 (57.4%)
Age at onset > 18 months, n (%)	40 (47.6%)
Incomplete KD, n (%)	39 (60.9%)
Complete KD, n (%)	28 (39.4%)
IVIG responders, n (%)	44 (53.0%)
IVIG non-responders, n (%)	17 (60.7%)
Summer, n (%)	13 (54.2%)
Other seasons, n (%)	54 (49.5%)

Legend: CALs stands for coronary artery lesions.

**Table 3:** Comparison of demographics, clinical data and echocardiographic findings recorded during the acute stage of KD between patients who developed CALs and those without coronary involvement.

Variable	No CALs	CALs	*p* Value
**Total**	382 (73.9%)	135 (26.1%)	
**Male gender, n (%)**	223 (70.5%)	93 (29.4%)	**0.031**
**Age**			
Mean age ± SD, months	44.1 ± 38.4	36.5 ± 34.1	**0.045**
Age at onset < 6 months, n (%)	30 (63.8%)	17 (36.2%)	0.093
Age at onset < 12 months, n (%)	70 (70.7%)	29 (29.3%)	0.395
Age at onset < 18 months, n (%)	95 (66.9%)	47 (33.1%)	**0.022**
**Ethnicity**			**0.014**
Caucasian, n (%)	342 (75.7%)	110 (24.3%)	
Asian, n (%)	12 (46.1%)	14 (53.9%)	
Afro-American, n (%)	15 (68.2%)	7 (31.8%)	
Mixed, n (%)	13 (76.5%)	4 (23.5%)	
**Seasonality**			0.290
Winter, n (%)	124 (71.3%)	50 (28.7%)	
Spring, n (%)	103 (77.4%)	30 (22.6%)	
Summer, n (%)	52 (68.4%)	24 (31.6%)	
Autumn, n (%)	102 (77.8%)	29 (22.1%)	
**Clinical presentation**			**0.010**
Complete, n (%)	256 (78.3%)	71 (21.7%)	
Incomplete, n (%)	126 (66.3%)	64 (33.7%)	
**IVIG responsiveness**			0.051
IVIG responders, n (%)	281 (77.2%)	83 (22.8%)	
IVIG non-responders, n (%)	57 (67.016.9%)	28 (32.9%)	
**Fever duration**			
Fever duration, mean ± SD	8.4 ± 3.9	10.8 ± 6.3	**<0.001**
Fever < 7 days	106 (80.9%)	25 (19.1%)	**0.032**
Fever > 7 days, n (%)	233 (71.5%)	93 (28.5%)	**0.037**
Fever > 10 days, n (%)	93 (64.1%)	52 (35.9%)	**0.001**
**Abdominal symptoms**	238 (73.9%)	84 (26.0%)	0.987
**Non-coronary cardiac involvement, n (%)**	78 (62.9%)	46 (37.0%)	0.001
**LV dysfunction, n (%)**	18 (66.7%)	9 (33.3%)	0.380
**Valvular regurgitation, n (%)**	24 (50%)	24 (50%)	**<0.001**
**Pericardial effusion, n (%)**	50 (68.5%)	23 (31.5%)	0.257

Legend: CALs stands for coronary artery lesions, SD stands for standard deviation, IVIG stands for intravenous immunoglobulin, LV stands for left ventricle.

**Table 4:** Laboratory findings in patients with and without CALs.

Variable	No CALs (n = 382)	CALs (n = 135)	*p* Value
**WBC × 10^9^ (/L), mean ± SD**	14.7 ± 5.9	14.8 ± 7.1	0.874
**N%, mean ± SD**	68.9 ± 15.4	68.7 ± 15.2	0.903
**L%, mean ± SD**	21.1 ± 12.7	21.7 ± 12.6	0.658
**RBC × 10^12^ (/L), mean ± SD**	4.3 ± 0.5	4.2 ± 0.6	0.087
**Hb (g/dL), mean ± SD**	11.1 ± 1.2	10.9 ± 1.4	**0.042**
**PLT × 10^9^ (/L), mean ± SD**	372.8 ± 182.7	376.6 ± 177.5	0.844
**Fibrinogen (mg/dL), mean ± SD**	569.9 ± 173.2	537 ± 174.7	0.347
**AST (UI/L), mean ± SD**	73.9 ± 25	62.1 ± 29	0.481
**ALT (UI/L), mean ± SD**	81.7 ± 42.1	68.7 ± 35.8	0.323
**AST/ALT**	1.33 ± 1.17	1.38 ± 0.95	0.742
**Albumin (g/dL), mean ± SD**	3.5 ± 0.7	3.2 ± 0.7	**0.008**
**CRP (mg/dL), mean ± SD**	9.2 ± 5.2	11 ± 5.4	**0.008**
**ESR (mm/h), mean ± SD**	63.9 ± 33.2	62 ± 31.1	0.701
**Na (mEq/L), mean ± SD**	134.5 ± 3.9	134.4 ± 3.2	0.837

Legend: WBC stands for white blood cells; N% stands for percentage of neutrophils; L% stands for percentage of lymphocytes; RBC stands for red blood cells; Hb stands for hemoglobin; PLT stands for platelets; AST stands for aspartate aminotransferase; ALT stands for alanine aminotransferase; CRP stands for C-reactive protein; ESR stands for erythrocyte sedimentation rate; Na stands for sodium.

**Table 5:** Multivariate analysis including variables independently associated with CALs.

Variable	OR	CI (95%)	*p* Value
**Fever > 10 days**	1.88	1.23–2.94	**0.004**
**Incomplete KD**	1.69	1.13–2.53	**0.010**
**Asian ethnicity**	4.97	1.16–5.75	**0.001**
**Age < 18 months**	1.63	1.06–2.51	**0.023**
**Male gender**	1.6	1.05–2.43	**0.026**

## Data Availability

The data presented in this study are available on request from the corresponding authors due to reasons of sensitivity.
